# A DROP-IN beta probe for robot-assisted ^68^Ga-PSMA radioguided surgery: first ex vivo technology evaluation using prostate cancer specimens

**DOI:** 10.1186/s13550-020-00682-6

**Published:** 2020-08-06

**Authors:** Francesco Collamati, Matthias N. van Oosterom, Micol De Simoni, Riccardo Faccini, Marta Fischetti, Carlo Mancini Terracciano, Riccardo Mirabelli, Roberto Moretti, Judith olde Heuvel, Elena Solfaroli Camillocci, Florian van Beurden, Henk G. van der Poel, Renato A. Valdes Olmos, Pim J. van Leeuwen, Fijs W. B. van Leeuwen, Silvio Morganti

**Affiliations:** 1 Istituto Nazionale di Fisica Nucleare, Sezione di Roma, Piazzale Aldo Moro 2, 00185 Rome, Italy; 2 Interventional Molecular Imaging Laboratory, Department of Radiology, Leiden University Medical Center, Albinusdreef 2, 2333ZA Leiden, The Netherlands; 3 Department of Urology, The Netherlands Cancer Institute—Antoni van Leeuwenhoek, Amsterdam, The Netherlands; 4 Dipartimento di Fisica, Università di Roma Sapienza, Piazzale Aldo Moro 5, 00185 Rome, Italy; 5 Dipartimento Di Scienze di Base Applicate per l’Ingegneria, Sapienza Università di Roma, Rome, Italy; 6 Scuola di specializzazione in Fisica Medica, Università Cattolica del Sacro Cuore, Rome, Italy; 7 Department of Nuclear Medicine, The Netherlands Cancer Institute—Antoni van Leeuwenhoek Hospital, Amsterdam, The Netherlands; 8 Scuola di specializzazione in Fisica Medica, Sapienza Università di Roma, Rome, Italy; 9 Section Nuclear Medicine, Department of Radiology, Leiden University Medical Center, Leiden, The Netherlands; 10 ORSI Academy, Melle, Belgium

**Keywords:** Radioguided surgery, Beta particle detection, Robot-assisted surgery, Prostate cancer, PET, PSMA

## Abstract

**Background:**

Recently, a flexible DROP-IN gamma-probe was introduced for robot-assisted radioguided surgery, using traditional low-energy SPECT-isotopes. In parallel, a novel approach to achieve sensitive radioguidance using beta-emitting PET isotopes has been proposed. Integration of these two concepts would allow to exploit the use of PET tracers during robot-assisted tumor-receptor-targeted. In this study, we have engineered and validated the performance of a novel DROP-IN beta particle (DROP-IN_β_) detector.

**Methods:**

Seven prostate cancer patients with PSMA-PET positive tumors received an additional intraoperative injection of ~ 70 MBq ^68^Ga-PSMA-11, followed by robot-assisted prostatectomy and extended pelvic lymph node dissection. The surgical specimens from these procedures were used to validate the performance of our DROP-IN_β_ probe prototype, which merged a scintillating detector with a housing optimized for a 12-mm trocar and prograsp instruments.

**Results:**

After optimization of the detector and probe housing via Monte Carlo simulations, the resulting DROP-IN_β_ probe prototype was tested in a robotic setting. In the ex vivo setting, the probe—positioned by the robot—was able to identify ^68^Ga-PSMA-11 containing hot-spots in the surgical specimens: signal-to-background (S/B) was > 5 when pathology confirmed that the tumor was located < 1 mm below the specimen surface. ^68^Ga-PSMA-11 containing (and PET positive) lymph nodes, as found in two patients, were also confirmed with the DROP-IN_β_ probe (S/B > 3). The rotational freedom of the DROP-IN design and the ability to manipulate the probe with the prograsp tool allowed the surgeon to perform autonomous beta-tracing.

**Conclusions:**

This study demonstrates the feasibility of beta-radioguided surgery in a robotic context by means of a DROP-IN_β_ detector. When translated to an in vivo setting in the future, this technique could provide a valuable tool in detecting tumor remnants on the prostate surface and in confirmation of PSMA-PET positive lymph nodes.

## Background

Radioguided surgery (RGS) is an interventional nuclear medicine technique that enables surgeons to identify, during the surgical procedure, lesions that had been detected with non-invasive preoperative imaging. Such guidance is achieved using a combination of radioactive tracers (i.e., radiopharmaceuticals) and intraoperative detection modalities [1]. The direct correlation between preoperative tracer mapping using, e.g., PET/CT and intraoperative detection, reduces the probability of missing a lesion that had been preoperatively identified using the imaging [2]. Applications of this approach include the localization of metastases or primary tumor margins [3].

Nowadays, a noticeable amount of commercially available radiopharmaceuticals is used for radioguided surgery [4]. Radioguidance based on low-energy (< 150 keV) gamma ray emitting radiopharmaceuticals is most commonly applied for sentinel lymph node (SN) biopsy procedures using (indocyanine green-)^99m^Tc-nanocolloid [5], radioguided occult lesion localization (ROLL) procedures using ^99m^Tc-labeled macro-aggregates [6], radioguided ^125^I-seed localization (RSL) procedures [7], and ^99m^Tc-PSMA-guided resection of lymph node metastases in prostate cancer patients [3]. Hence, the most frequently used detection modality for intraoperative localization is the gamma-detection probe, which provides numerical and acoustical feedback proportional to the amount of radiopharmaceutical localized. Unique for this modality is that it supports relatively “deep” signal detection (i.e., tissue only provides marginal attenuation of gamma ray emissions). Recently, the introduction of the DROP-IN gamma (DROP-IN_γ_) probe concept helped to make radioguidance compatible with robotic surgery [8–11].

For many diagnostic evaluations (e.g., PSMA), PET radiopharmaceuticals are still preferred. These PET isotopes induce both gamma ray from annihilation (i.e., 511 keV photon) and β+ particle (i.e., positron) emissions, providing two possible detection routes that can be exploited for radioguidance purposes. Since the intraoperative detection of 511 keV gamma rays requires heavily collimated approaches, and thus cumbersome probes, direct detection of β+ particle emissions has been explored [4]. Recently, a sensitive β-probe detector-technology, appropriate for both β− (i.e. electron) and β+ radioguided surgery was introduced [12–14]. Due to intrinsic differences among the interaction with matter of beta and gamma particles, beta probes require a small active area and basically no collimation: as a result, such β-probes can be much smaller and lighter than γ-probes, especially when active materials are chosen that are insensitive to the 511 keV γ-ray background [15]. Therefore, such a detector allows to exploit the unique spatial resolution achievable with beta emission. In fact, tissue penetration of ~ 1 MeV β-particles is much smaller than that of γ-rays (~ millimeters vs ~ centimeters) making it a unique “surface scanning” technique, much less limited by the “shine-through” of deeper lying tracer uptake [12]. Direct beta detection might thus be a very effective methodology to detect tumors nearby healthy organs characterized by elevated physiological uptake of the radiopharmaceutical (e.g., tumor nearby healthy prostate).

**Fig. 1:**
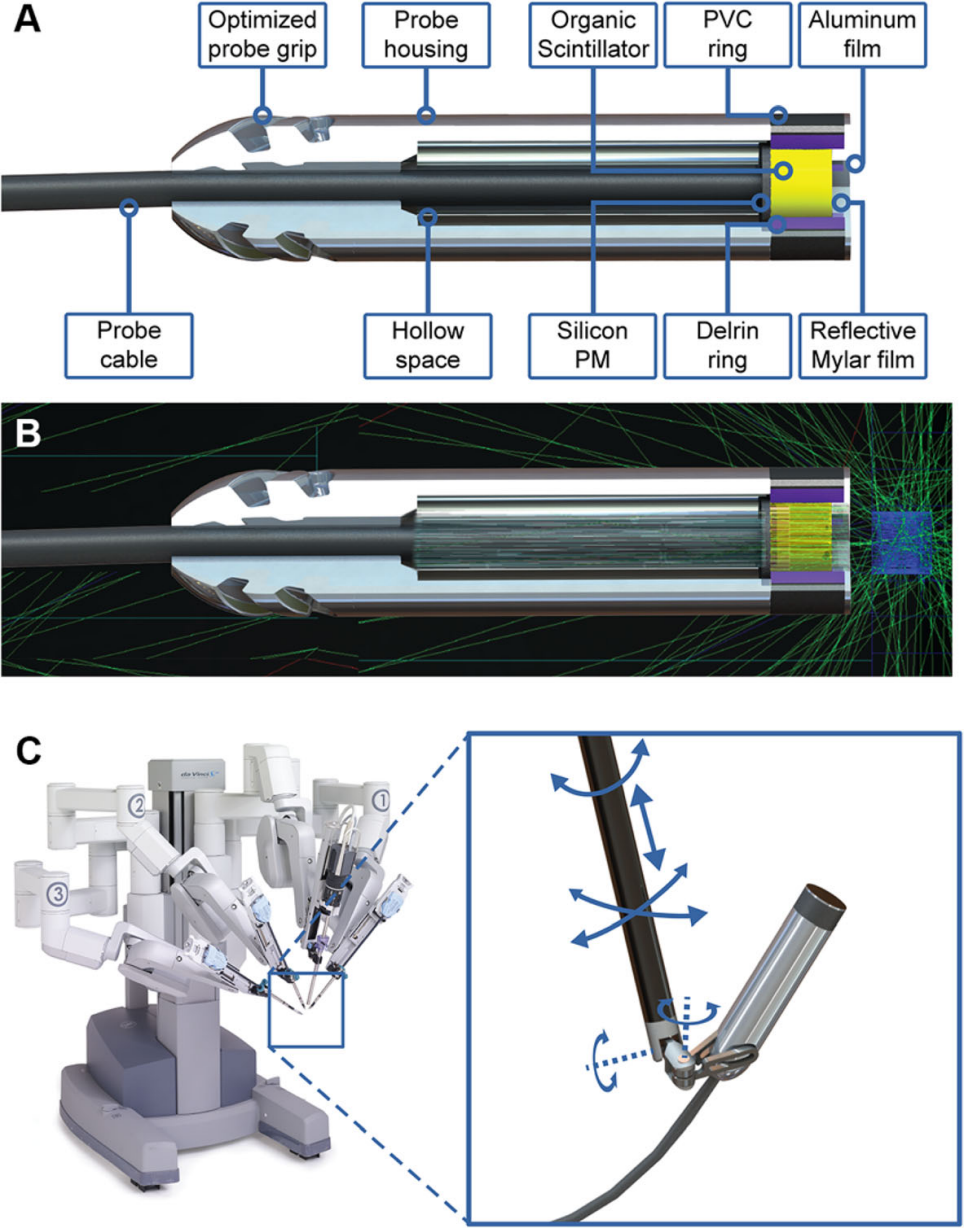
DROP-IN_β_ probe design. **a** Schematic representation of the probe components. **b** Example of one of the Monte Carlo simulations optimizing β-particle detection and γ-photon transparency. **c** Overview of the probe application setup, showing its high maneuverability

In an effort to explore the use of the widely available PET radiopharmaceutical [^68^Ga]Ga-PSMA-11 (^68^Ga-labeled Glu-urea-Lys (Ahx)-HBED-CC) for robot-assisted radioguided surgery purposes, we have developed a DROP-IN beta (DROP-IN_β_) probe that exploits both the high beta detection efficiency and the compactness of such a detector [12] with the maneuverability of the DROP-IN concept [11] (see Fig.1). In this paper, we present its engineering together with its first characterization on ex vivo surgical specimens (i.e., prostate and lymph nodes) of PSMA-PET positive patients that received an additional dose of [^68^Ga]Ga-PSMA-11 during surgery.

## Methods

### DROP-IN_β_ probe development

The β-detection probe used in this study was based on a cylindrical scintillator (6 mm diameter and 3 mm height) made of mono-crystalline para-terphenyl (doped with 0,1% in mass of (E,E)-1,4-Diphenyl-1,3-butadiene) [15]. Being a non-hygroscopic organic scintillator with high light yield (~ 140% of anthracene) and low density (1.23 g/cm^3^), this material provides a high sensitivity to β particles and elevated transparency to photons (e.g., the 511 keV γ rays as induced by PET radiopharmaceuticals). To improve light collection from the scintillator, the detector was surrounded with a 2-mm-thick white diffusing Delrin ring and covered in front with two 4 μm layers of a reflective aluminized-Mylar film. The light tightness of this assembly was achieved by adding an external black poly-vinyl-chloride ring of 2 mm, covered on the front by a 15-μm layer of aluminum. Light collection efficiency was maximized using a 3 × 3 mm^2^ silicon photomultiplier (SiPM C-series 30035, SensL Ltd.). After a first Monte Carlo-based study of such a probe in a Ga-PSMA context [16], a dedicated laboratory characterization has been performed. A detection efficiency of ~ 90% for ^68^Ga β particles and ~ 2.5% for 511 keV γ rays has been found [17].

The β detector was placed at the tip of the DROP-IN probe housing. Similarly to the previously optimized DROP-IN_γ_ probe [11], a 45° angle grip was incorporated at the end of longitudinal axis of the probe, tailored to the ProGrasp Forceps (Intuitive Surgical Inc.), an instrument that is often used during a prostatectomy and lymph node dissection. Maintaining its compatibility with the da Vinci (Intuitive Surgical Inc.) apparatus, this ensured the maneuverability needed to fully exploit the specificity of beta-RGS. In fact, differently from gamma probes, beta detection requires the probe to have full access to the surface to be examined, due to the significant signal attenuation in tissue.

The housing was printed using acrylonitril-butadieen-styreen plastics and a Dimension Elite 3D printer (Stratasys Ltd.). Final dimensions of the whole probe were a length of 55 mm and a diameter of 12 mm, due to the available detector prototype. In the future, however, this diameter could be reduced (e.g., to 8 mm) if necessary.

Portable electronics based on an Arduino Due (Arduino AG) equipped with a custom analog shield providing signal conditioning and trigger logic were used for the readout [18]. Sampling time was 1 s. At the end of the chain, the output in terms of counts per second (CPS) was displayed on a tablet, via wireless connection.

### Optimization of the DROP-IN_β_ probe design

In order to optimize the design of the β-probe, a dedicated Monte Carlo simulation was performed in Geant4 [19]. In this simulation, the whole detector was reconstructed, and all physical processes of interest were taken into account to effectively reproduce particle scattering, absorption, energy deposition, and secondary particles generation. These simulations indicated that a cavity behind the β particle detector would result in a lower noise-background: additional layers of material could in fact promote β+ to γ conversion close to the detector, creating noise-background (Fig. 1b). This design concept yielded a light-weight probe construction (Fig. 1a), mostly transparent to 511 keV **γ**-induced noise.

### First ex vivo probe evaluation

#### Patient selection

In total, 7 patients with primary diagnosed locally (advanced) high-risk prostate cancer were included (see Table 1). Inclusion criteria consisted of a primary tumor ≥ 2 cm (based on MRI) with a minimal average PSMA tracer uptake of 1.7 kBq/mL (based on PSMA PET/CT). These patients were mostly redirected to our clinical institute for prostate cancer treatment; initial diagnostics was performed at the referring hospital. Therefore, based on local availability and preferences, diagnostic PSMA-PET/CT was performed with ^18^F-DCFPyl. This should however provide comparable uptake as ^6^[^68^Ga]Ga-PSMA-11 [20]. SUV_mean_ measurements were performed by manually defining a volume of interest in the prostate tumor, using OsiriX medical imaging software (Pixmeo SARL). All patients were scheduled for a robot-assisted radical prostatectomy and extended pelvic lymph node dissection. In order to minimize radioactive exposure to both patient and medical personnel, a limited dose of ~ 70 MBq (median 68, IQR 63.5–82) [^68^Ga]Ga-PSMA-11 for radioguidance was intravenously administered in the operating room (OR), after docking the da Vinci robot. The study was approved by the local ethics committee (NL66218.031.18, trial NL8256 at trialregister.nl) and all patients provided a written informed consent.

**Table 1:** Preoperative patient characteristics

Pt #	Age	PSA (ng/mL)	Gleason score	Prostate volume on MRI (cc)	Tumor stage	SUV_mean_ in primary tumor focus on PET	SUV_mean_ positive LNs on PET
**1**	71	4.4	4 + 4 = 8	30	cT2aN0M0	13.8	N.A.
**2**	57	5.3	4 + 4 = 8	55	cT1cN0M0	3.3	N.A.
**3**	73	8.3	4 + 5 = 9	76	cT3aN1M0	17.8	5.6 (ExR), 3.1 (ObR)
**4**	66	2.7	4 + 4 = 8	47	cT3bN0M0	4.1	N.A.
**5**	63	6.4	4 + 5 = 9	41	cT2cN0M0	11.7	N.A.
**6**	55	9.3	4 + 4 = 8	28	cT2bN0M1	14.7	N.A.
**7**	48	4.4	4 + 5 = 9	62	cT3bN1M0	13.3	4.8 (ObL), 3.5 (ExR)

*Pt #* patient number, *N.A.* not applicable, *LNs* lymph nodes, *ExR* external iliac right, *ObR* obturator right, *ObL* obturator left

#### Probe countings

At the end of the surgical procedure, roughly 2.5 h after injection (median 150 min; IQR 120–172.5), the surgical specimens (prostate and lymph node packages if present) were rinsed with saline and scanned using the DROP-IN_β_ probe mounted on a da Vinci robot using the ProGrasp forceps instrument. Rinsing of the ex vivo specimens was performed to remove possible urine contamination, since [^68^Ga]Ga-PSMA-11is known to undergo renal clearance [20]. For prostate samples, “signal” was defined as the highest counting area, as confirmed with preoperative imaging information. The “background” was defined as the area nearby the “signal” where the counting rate dropped to the plateau value that was found in the rest of the sample (thus representing tracer uptake in the healthy prostate tissue). For lymph node samples, the “signal” was acquired on the lymph node itself, and “background” on the surrounding tissue (i.e., fat tissue and negative lymph nodes).

#### Pathology

Following analysis, all specimens were sent to pathology for assessment using standard histopathological procedures [21]. Additionally, distances between the tumor and the inked specimen borders were measured at marked locations.

#### Monitoring of radioactive exposure in the operating room

To investigate the feasibility of radioguided surgery using [^68^Ga]Ga-PSMA-11, radiation safety was considered an important topic. Therefore, radiation dose, as received by the operating room staff, was carefully monitored [22]. The surgeon (located behind the robotic console), the scrub nurse (located next to the patient in the sterile field), the assisting nurse (moving around the operating room, outside the sterile field), the anesthetist (located at the head of the patient, outside the sterile field), and the researcher (located > 1 m away from the patient, outside the sterile field) all had their own electronic radiation dosimeter (MGPInstruments DMC 2000; Mirion Technologies, Ltd.).

## Results

### Probe usage

The developed DROP-IN_β_ probe easily fitted through standard 12 mm trocars and pick-up of the probe was facile using the standard da Vinci instruments of the surgical robot. Being a tethered design, probe maneuverability allowed for positioning with 6 degrees of freedom, as inherited from the ProGrasp forceps, with an effective scanning range of 0–140^**o**^ around the tip of the instrument. Scanning with the probe could be performed autonomously from the surgical robotic console, not requiring the help of an assistant.

### Ex vivo probe evaluation

**Fig. 2:**
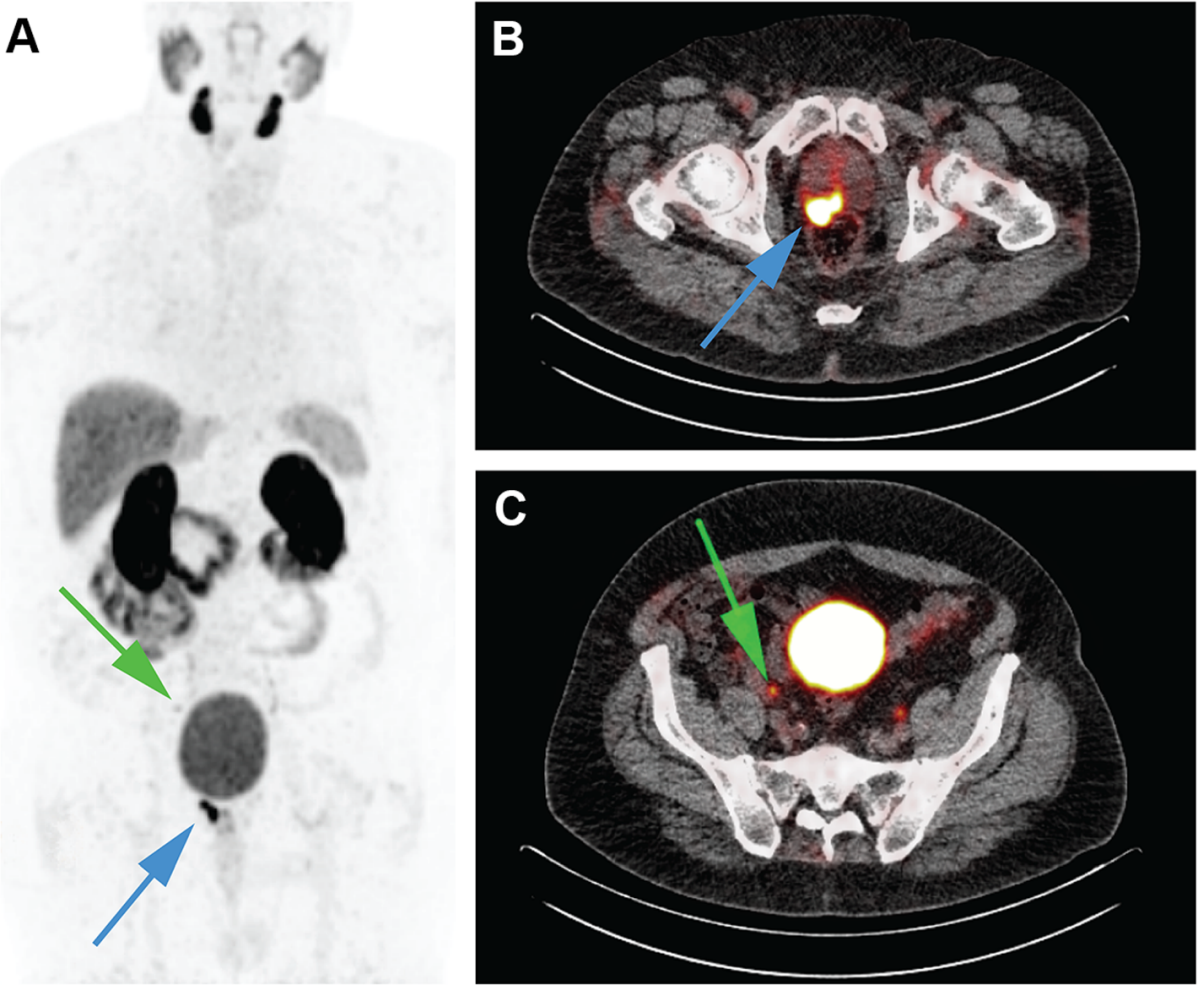
Preoperative tumor mapping using PSMA-PET. **a** Example of total body PET maximum intensity projection with tumor focus in prostate (blue, upwards arrow) and lymph node metastasis (green, downwards arrow). **b** PET/CT slice of the same patient illustrating a clear tumor focus within the prostate (blue arrow; SUV_mean_ = 17.8). **c** PET/CT slice of the same patient displaying a lymph node metastasis (green arrow; SUV_mean_ = 5.6)

The seven included patients displayed clear PSMA-PET positive primary tumors (see Table 1), with a SUV_mean_ in the tumor > 3. Additionally, two patients had PSMA-PET positive lesions, suspected for lymph node metastases (see Fig. 2 and Table 1).

**Fig. 3:**
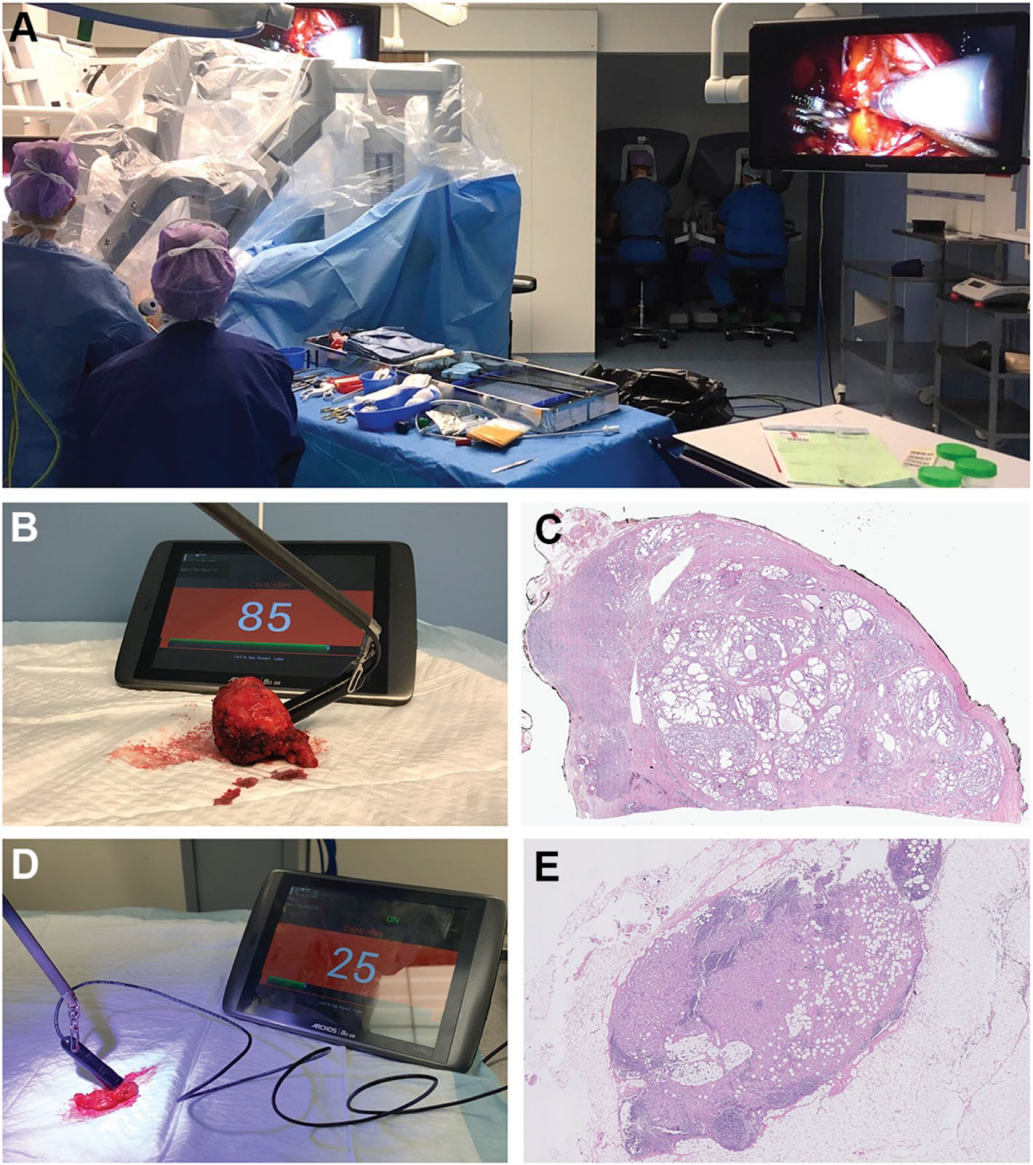
DROP-IN_β_ probe evaluation in relation to pathology. **a** Overview of the robot-assisted OR setup. **b** Example of robot-assisted beta-tracing with the DROP-IN_β_ probe on the surface of a resected prostate sample. **c** Histopathology slide displaying tumor spread within the prostate with respect to the specimen surface. **d** Example of robot-assisted beta-tracing on the surface of a resected lymph node package. **e** Histopathology slide showing tumor spread within a PSMA-PET positive lymph node

Figure 3 illustrates usage of the DROP-IN beta probe on surgical specimens using the da Vinci robot. Table 2 shows a summary of the collected data. In general, probe background measurements without any tissue (i.e., “dark counts”) were in the order of 0–2 CPS, while uncovered tumor areas, cleaved if necessary, provided count rates between 130 and 250 CPS. Due to its normal (i.e., default) PSMA expression levels, healthy prostate tissue yielded ~ 5–45 CPS. The primary tumor in patients 1, 3, 5, 6, and 7 provided a maximum S/B (signal to background ratio) > 5, displaying a maximum count rate of ~ 247 CPS on the surface of the excised prostate specimen. At pathology, only patients 1 and 7 harbored true positive resections margins (i.e., tumor cells were found in the inked borders of the prostate at pathology). However, in patients 3, 5, and 6, tumor was found within 1 mm of the resection margin, confirming a superficial tumor location. The maximum S/B measured for the prostate specimens in patients 2 and 4 was much lower, < 2.5. In these cases, pathology indicated that the tumor was located > 1.5 mm below the specimen margin, indicating a negative surgical margin. This occurrence, together with their smaller SUV_mean_ with respect to other cases, ended up limiting as expected the possibility of beta-tracing.

**Table 2:** Probe evaluation in relation to pathology

Pt #	Probe evaluation				Pathologic evaluation				
	Injected activity [MBq]	Total activity left at time of scanning [MBq] and injection-surgery time interval [min]	S/B prostate tumor*	S/B PET positive LNs	Extra-capsular tumor spread	Positive resection margins	Shortest tumor-border distance [mm]	Tumor in PET Positive LNs	Tumor stage
**1**	68	11 (180)	107/14 = 7.6	N.A.	Yes	Yes	0	N.A.	pT3aN0 R1
**2**	88	15 (170)	40/16 = 2.5	N.A.	No	No	> 3	N.A.	pT2N0 R0
**3**	76	13 (175)	108/15 = 7.2	50/8 = 6.3 (ExR), 34/10 = 3.4 (ObR)	Yes	No	< 1	Yes (ExR), no (ObR)	pT3bN1 R0
**4**	97	32 (110)	88/40 = 2.2	N.A.	Yes	No	> 1.5	N.A.	pT3bN0 R0
**5**	65	14 (150)	108/15 = 7.2	N.A.	No	No	< 0.5	N.A.	pT2N0 R0
**6**	62	18 (120)	247/45 = 5.5	N.A.	Yes	No	< 0.5	N.A.	pT3aN1 R0
**7**	23	7 (120)	35/7 = 5.0	20/3 = 6.7 (ObL), 9/3 = 3.0 (ExR)	Yes	Yes	0	Yes(ObL), yes (ExR)	pT3bN1 R1

*Pt #* patient number, *S/B* signal to background, *N.A.* not applicable, *ExR* external iliac right, *ObR* obturator right, *ObL* obturator left

*As measured on the surface of the resected specimen. PET negative lymph nodes that were excised are not added in the table for clarity sake, since no sign of elevated tracer uptake was found in none of them with the probe either

Interestingly, patients 3 and 7 both harbored 2 lymph nodes each that were positive on preoperative PSMA-PET. Using the DROP-IN_β_ probe, these lymph nodes also showed elevated tracer uptake with respect to the other lymph nodes and surrounding fat tissue: S/B > 3. At pathology, metastasis was only found in three of these lymph nodes, suggesting a false-positive PSMA uptake in one lymph node. In this limited group of PET positive lymph nodes, the smallest metastasis the probe was capable to detect had a 7-mm diameter (SUV_mean_ of 5.6 on preoperative PSMA-PET, time between injection and measurement 3 h). All PET negative lymph nodes that were excised and analyzed yielded the same counting rates as nearby background tissue.

### Monitoring of radioactive exposure in the operating room

The average radiation dose per surgery performed, as measured for the operating room staff, was 0.005 mSv for the surgeon, 0.016 mSv for the scrub nurse, 0.002 mSv for the assisting nurse, 0.001 mSv for the anesthetist, and 0.001 mSv for the researcher. Taking in to account the guidelines from the International Commission on Radiological Protection [23], this would mean that the surgeon would be allowed to perform 200 of such [^68^Ga]Ga-PSMA-11 guided procedures a year, while the scrub nurse would be limited to 62 procedures a year.

## Discussion

In this study, the first steps are made towards integration of beta radioguided surgery within the robot-assisted setting. Using the DROP-IN concept, the surgeon has full control of probe placement, yielding autonomy and great maneuverability during radioguidance [8–11]. Direct beta detection provides, thanks to its specificity and sensitivity, a useful way to probe prostate margins and suspect lymph nodes.

This initial ex vivo validation of the DROP-IN_β_ probe concept showed a high signal to background (> 5) for tumors located < 1 mm from the resected surface, suggesting that the technique has the potential to support robotic surface scanning of primary tumor margins in prostate cancer. Even more precise characterization of the possible lesion depth with respect to the surgical margin might be possible with future developments in the underlying detection software algorithms [24]. In addition, confirming PSMA-positive lymph nodes (S/B > 3), the DROP-IN_β_ probe concept might also support the intraoperative identification of metastatic lymph nodes.

Compared to the previously reported use of a DROP-IN_γ_ probe in combination with the tracer [^99^Tc]Tc-PSMA-I&S (i.e., salvage procedures for lymphatic metastases) [10], the use of a DROP-IN_β_ probe in combination with [^68^Ga]Ga-PSMA-11 possesses some unique advantages. First of all, this approach supports the use of more widely available PET tracers. Secondly, the limited tissue penetration of β particles (only a few millimeters) allows for an accurate surface scanning of the primary tumor margins [12], thus highlighting possible tumor localizations on the prostate surface. Indeed, in the current study, beta radiation was severely attenuated when > 1.5 mm of healthy tissue was located between the surface of the prostate specimen and the pathological tumor margins. In this sense, β-tracing benefits from similar positive features as fluorescence imaging [25], i.e., no ‘shine-through’ of neighboring or deeper lying tracer uptake and a superior spatial resolution [12, 26]. These features are essential when the extra-capsular spread of PSMA-overexpressing tumor lesions is pursued in a prostate with (significant) default PSMA expression [27]. Consequently, β-tracing could provide a superior means for margin assessment during, e.g., nerve sparing surgery [28, 29]. Alternative to investigated beta-radiation detection for tumor margin assessment on the prostate surface, fully matured ex vivo technologies are available (e.g., NeuroSAFE [30]) and alternative β-emission-based imaging technologies are being explored (e.g., Cerenkov [22]*)*. Future research, and in particular randomized trials, will have to show which technology is superior, or if different technologies can work in synergy.

Potential limitations of the proposed [^68^Ga]Ga-PSMA-11-guided surgery concept are the radiation dose for the surgical staff (currently limited to about 62 procedures a year) and the contamination of the prostate margins by tracer containing urine. The DROP-IN_β_ probes ability to detect lesions using < 70 MBq doses helps limit the exposure of the surgical staff. It is worth highlighting in particular that injecting the radiotracer directly in the operating room allowed ex vivo examination after ~ 2.5 × *t*_1/2_ (3 h, *t*_1/2_ = 68 m). Hence future in vivo application, e.g., 1 h p.i., would allow an even lower activity to be used to achieve a similar detection sensitivity, namely of the order of 40 MBq. Regarding the urine contamination of the samples, as stated previously, the accumulation of PSMA tracers in healthy organs and in particular urine may yield background signals that complicate intraoperative margin detection [20]. However, the direct detection of beta particles performed with a detector substantially transparent to gamma rays, as suggested in this paper, should drastically reduce the impact of such a background; only the signal originating from a few millimeters around the detector should be detected (i.e., thus only a small urine layer must be considered [24, 31]). Nonetheless, acknowledgement of this effect by radiochemists [32, 33] and the reduced renal clearance of for example [^18^F]F-PSMA tracers [34, 35] may in the future help to overcome these issues. In addition, the influence of renal clearance might also be overcome by using β-emitting isotopes that have a longer half-life, allowing the tumor resection to take place after all renal clearance of non-bound tracer is realized, e.g. using alternative PET isotopes such as ^64^Cu (*t*_1/2_ = 12.7 hours), or even theranostic isotopes such as ^67^Cu (*t*_1/2_ = 2.5 days), ^90^Y (*t*_1/2_ = 2.66 days), or ^177^Lu (*t*_1/2_ = 6.6 days) [36, 37].

## Conclusion

In this study, we presented the integration of two recent developments in RGS: a high efficiency beta detector and a flexible DROP-IN probe housing compatible with robot-assisted surgery. The first prototype of DROP-IN_β_ probe has been successfully validated on ex vivo samples of prostate tumors with [^68^Ga]Ga-PSMA-11, being able to detect all PET positive resected specimens, with a smallest detected dimension in this data sample of 7 mm. Probe maneuverability was found to be the same of the DROP-IN_γ_ concept, which has already demonstrated its efficacy in in vivo tests. This DROP-IN_β_ probe could thus help exploit the growing amount of disease specific PET tracers and may help provide a new powerful tool to perform tumor margin evaluation and confirm metastatic spread.

## Data Availability

The Monte Carlo simulation datasets used during the current study are available from the corresponding author on reasonable request. All data gained on patient samples during this study are included in this published article.

## Abbreviations

RGS: Radioguided surgery
PSMA: Prostatic Specific Membrane Antigen
S/B: Signal to background ratio
OR: Operating room
